# Case Report: Successful treatment of dystrophic epidermolysis bullosa pruriginosa with upadacitinib in a patient with *COL7A1*, *CARD14*, and *G6PD* gene mutations

**DOI:** 10.3389/fmed.2026.1805186

**Published:** 2026-05-08

**Authors:** Hao Li, Yu Zhang, Xuewen Lin, Tianmeng Yan, Xiaoyan Wu, Zhenying Zhang

**Affiliations:** Department of Dermatology, The University of Hong Kong–Shenzhen Hospital, Shenzhen, China

**Keywords:** *CARD14*, case report, *COL7A1*, dystrophic epidermolysis bullosa pruriginosa, *G6PD*, genetic variant, JAK inhibitor, upadacitinib

## Abstract

Dystrophic epidermolysis bullosa pruriginosa (DEB-Pr) is a rare subtype of dystrophic epidermolysis bullosa (DEB) characterized by intensely pruritic blisters, prurigo-like nodules, and scarring on the extensor aspects of the extremities. We present a 43-year-old female patient with a 30-year history of recurrent erythematous plaques, nodules, and blisters, which was accompanied by severe pruritus affecting the scalp, back, and extremities. Whole-exome sequencing identified a heterozygous likely pathogenic variant in *COL7A1* (c.6760G>A, p.Gly2254Arg), a heterozygous variant of uncertain significance (VUS) in *CARD14* (c.2172C>A, p.Tyr724*), and a heterozygous pathogenic variant in *G6PD* (c.1388G>A, p.Arg463His). Combined with the patient’s clinical manifestations, histopathological findings, direct immunofluorescence results, negative results for pemphigus and pemphigoid autoantibodies, genetic testing results, and past medical history, the patient was diagnosed with refractory DEB-Pr and glucose-6-phosphate dehydrogenase (G6PD) deficiency. To the best of our knowledge, the co-occurrence of these three distinct variants in a patient with DEB-Pr has not been previously reported in the literature. Oral upadacitinib (initiated at 15 mg once daily) induced rapid and substantial relief of pruritus as well as improvement of cutaneous lesions. Clinical symptoms continued to improve during subsequent gradual dose tapering, and maintenance therapy with 15 mg once weekly has been administered since month 16 of treatment, with a 7-month follow-up to date. No treatment-related adverse events were observed throughout the entire 22-month follow-up period. This case suggests that Janus kinase (JAK) inhibitors may represent a feasible therapeutic option for patients with similar refractory DEB-Pr.

## Introduction

Epidermolysis bullosa (EB) encompasses a group of rare inherited mucocutaneous disorders caused by pathogenic variants in genes encoding structural proteins of the dermal-epidermal junction, resulting in blister formation following minor mechanical trauma (1). Dystrophic epidermolysis bullosa pruriginosa (DEB-Pr), a rare subtype of DEB linked to *COL7A1* variants, is clinically characterized by intensely pruritic blisters, prurigo-like nodules, and scarring on the extensor aspects of the extremities (1). Diagnosis is typically established by a combination of clinical manifestations, histopathological examination, and comprehensive genetic testing. Conventional treatments, including topical corticosteroids, oral antihistamines, and thalidomide, yield only limited clinical benefit (2). Recent studies have highlighted biological agents and small-molecule inhibitors as promising therapeutic strategies for this condition.

### Case description

**Figure 1:**
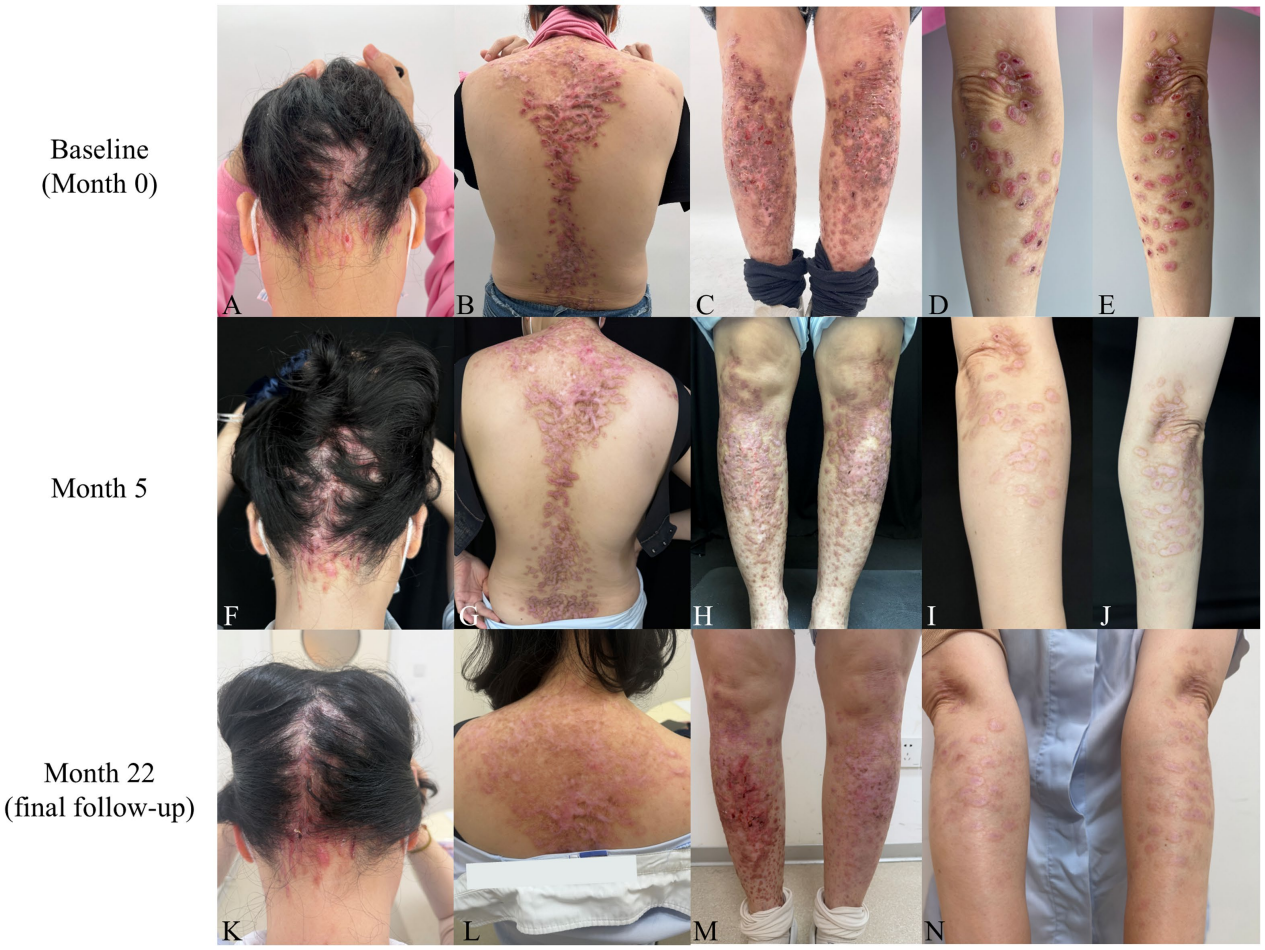
Clinical evolution of cutaneous lesions in a patient with DEB-Pr treated with upadacitinib. **(A**–**E)** Baseline (Month 0): Prior to upadacitinib initiation, multiple dark-red nodules, blisters, and scar-like plaques were noted on the scalp, back, and extensor aspects of the extremities, with partial confluence. Erosions, excoriations, and crusting were visible on some lesions, and pruritus was severe (VAS 9). **(F**–**J)** Month 5 of treatment: Following 3 months of upadacitinib 15 mg once daily and 2 months of 15 mg every other day, cutaneous lesions were markedly flattened and faded, with residual post-inflammatory hyperpigmentation. Pruritus was mild and intermittent (VAS 1–2). **(K**–**N)** Final follow-up (Month 22): The patient had received maintenance therapy with upadacitinib 15 mg once weekly for 7 months. Most original lesions had resolved with residual mild scarring; scattered excoriations and crusting were present on the scalp and right lower leg, and pruritus was mild and intermittent (VAS 1–2).

**Figure 2:**
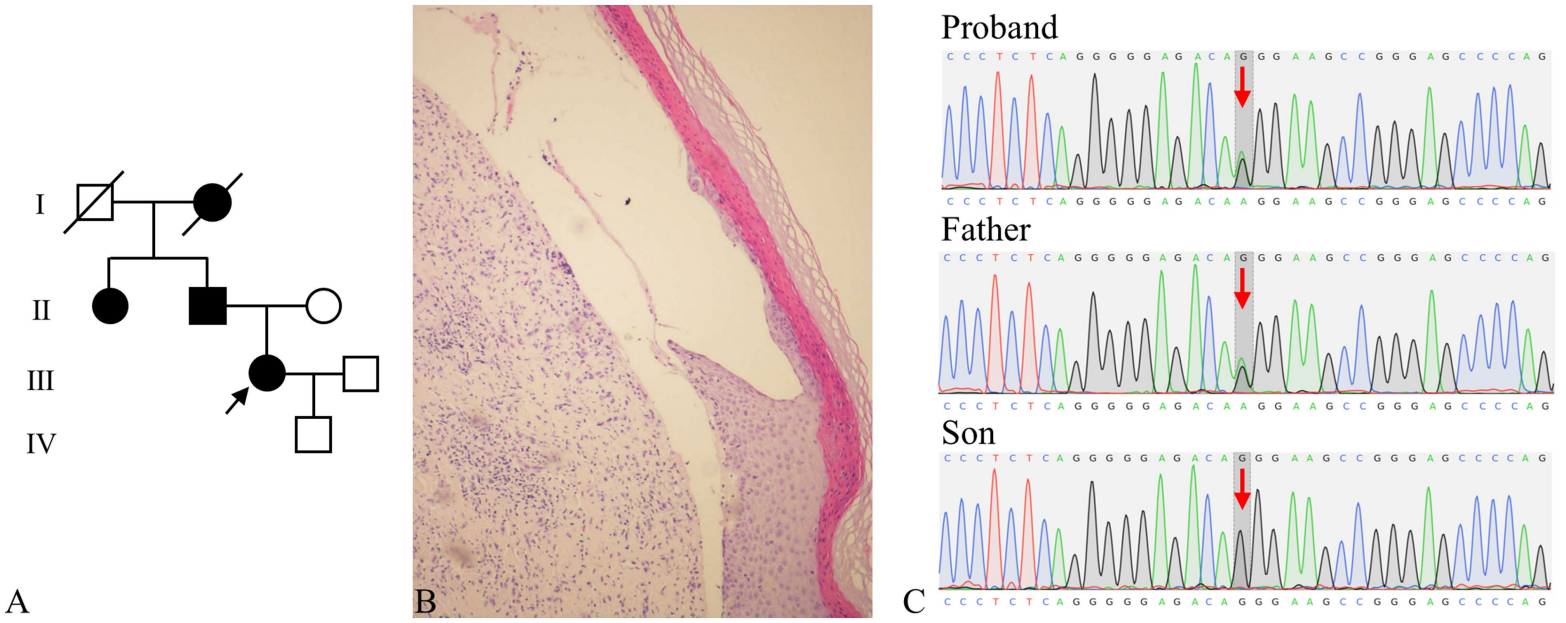
**(A)** Pedigree analysis demonstrating an autosomal dominant inheritance pattern. The arrow indicates the proband; filled symbols represent affected individuals (circles = female, squares = male). **(B)** Histopathological features of DEB-Pr (hematoxylin and eosin staining): Hyperkeratosis, parakeratosis, and focal acanthosis, and subepidermal clefts with a sparse lymphohistiocytic infiltrate. **(C)** Sanger sequencing chromatograms confirmed a heterozygous *COL7A1* variant (NM_000094.4: c.6760G>A, p.Gly2254Arg) in the proband and her father, while her son exhibited the wild-type *COL7A1* genotype.

A 43-year-old female presented with a 30-year history of erythematous plaques, nodules, and blisters associated with severe pruritus involving the scalp, back, and extremities. Intense pruritus led to repeated scratching, resulting in erosion and crusting of lesions, with subsequent thickening of the affected skin. Intermittent topical corticosteroid cream application in the past had shown poor efficacy, and cutaneous lesions gradually enlarged due to persistent severe pruritus and repetitive scratching. The patient presented to our department in February 2024, and treatment with regular topical corticosteroids combined with oral antihistamines failed to alleviate symptoms (Figures 1A–E). Severe nocturnal pruritus significantly impaired her sleep quality. The pruritus Visual Analog Scale (VAS; 0–10, 0 = no pruritus, 10 = worst imaginable pruritus) score was 9. Her past medical history was significant for *G6PD* deficiency (favism). The patient’s father had a history of erythematous patches, blisters, and scarring on the trunk and extremities. The patient reported that her paternal aunt and paternal grandmother had presented with similar cutaneous manifestations, although detailed clinical information was unavailable (Figure 2A).

Skin biopsy of lesions on the patient’s upper extremities was performed in March 2024. Histopathological examination revealed hyperkeratosis, parakeratosis, and focal acanthosis with elongated rete ridges, subepidermal clefts containing a sparse infiltrate of lymphocytes and histiocytes, degeneration of superficial dermal collagen, fibroblast and small vascular proliferation, and perivascular lymphocytic infiltration with occasional eosinophils (Figure 2B). Direct immunofluorescence staining for IgG, IgA, IgM, and C3 was uniformly negative. Circulating autoantibodies against type XVII collagen (BP180), bullous pemphigoid 230 kDa protein (BP230), desmoglein-1, and desmoglein-3 were undetectable. Whole-exome sequencing was performed on the proband, her father, and her son, with the following results: (a) A heterozygous likely pathogenic variant (PM2 + PP3) in *COL7A1* (NM_000094.4: c.6760G>A, p.Gly2254Arg) was detected in both the proband and her father, but was absent in her son (Figure 2C). (b) A heterozygous VUS in *CARD14* (c.2172C>A, p.Tyr724*) was identified in the proband, but not in her father or son. (c) The proband harbored a heterozygous pathogenic variant (PS3 + PS4 + PP3 + PP4) in *G6PD* (c.1388G>A, p.Arg463His), while her father and son were hemizygous for this pathogenic variant (3).

Based on the comprehensive medical history, clinical manifestations, family history, histopathological findings, immunological results, and genetic testing results, the patient was diagnosed with DEB-Pr complicated by *G6PD* deficiency. Baseline laboratory investigations completed prior to treatment (May 20, 2024) revealed a hemoglobin (Hb) of 99 g/L, triglycerides (TG) of 2.03 mmol/L, LDL-C of 4.00 mmol/L, HDL-C of 1.10 mmol/L, and total cholesterol (TC) of 5.37 mmol/L, indicating anemia and dyslipidemia in the patient. Following written informed consent, oral upadacitinib was initiated on May 27, 2024, at a dose of 15 mg once daily. Dose adjustments and clinical responses over the 22-month treatment period are detailed as follows:

- *Period 1 (Months 0–3, May–August 2024)*: Upadacitinib 15 mg once daily was administered, resulting in rapid and significant pruritus relief. The VAS score decreased from 9 to 4 within 48 h and further declined to 1 at 3 months. Excoriations and erosions had completely healed within 1 month, with no new blisters or crust formation.
- *Period 2 (Months 4–10, September 2024–March 2025)*: The dose was tapered to 15 mg every other day. Mild recurrent pruritus (VAS 1–2) was noted, with no exacerbation of cutaneous lesions. Lesions continued to flatten and fade, with residual post-inflammatory hyperpigmentation (Figures 1F–J).
- *Period 3 (Months 11–15, April–August 2025)*: Dosing was further adjusted to 15 mg every third day. Mild pruritus persisted (VAS 1–2), and pre-existing lesions continued to improve. A small number of new scratch-induced nodules and excoriations developed on the scalp and lower legs.
- *Period 4 (Months 16–22, September 2025–March 2026)*: Maintenance therapy with 15 mg once weekly was initiated. Mild intermittent pruritus (VAS 1–2) persisted, with no exacerbation of the original cutaneous lesions. Self-discontinuation of treatment for 2 weeks at month 19 (December 2025) led to pruritus recurrence (VAS 4–6) and the occurrence of new nodules and excoriations on the lower limbs. Resumption of weekly upadacitinib dosing promptly halted the development of new lesions. At the final follow-up (month 22), mild intermittent pruritus (VAS 1–2) was still present. Most original lesions had resolved, with residual mild scarring, while scattered new nodules and excoriations were noted on the scalp and right lower leg (Figures 1K–N).

Throughout the entire 22-month treatment and follow-up period, regular laboratory monitoring was performed, including complete blood count, creatine kinase, D-dimer, lipid profile, liver function tests, and renal function tests. In the absence of other systemic pharmacotherapy, baseline anemia gradually improved (Hb 137 g/L on December 29, 2025), and dyslipidemia was ameliorated (TG 1.40 mmol/L, LDL-C 3.67 mmol/L, HDL-C 1.18 mmol/L, TC 5.08 mmol/L on December 29, 2025). No other significant laboratory abnormalities were detected throughout the follow-up period. No treatment-related adverse events, including infection or thromboembolic events, were observed. The patient remains under regular follow-up to date.

## Discussion

Epidermolysis bullosa (EB) is a group of inherited monogenic disorders caused by pathogenic variants in more than 20 genes encoding key structural proteins of the basement membrane zone (4). These variants result in dermal-epidermal separation, persistent blisters, and erosions in patients with mechanically fragile skin following minor trauma. Based on the affected structural proteins and the corresponding level of skin fragility and blistering, EB is classified into four major subtypes: epidermolysis bullosa simplex (EBS), junctional EB (JEB), dystrophic EB (DEB), and Kindler EB (KEB) (5).

Dystrophic epidermolysis bullosa (DEB) is a rare subtype characterized by bulla formation below the dermal-epidermal junction in the papillary dermis, caused by pathogenic variants in *COL7A1* (chromosome 3p21.1), which encodes the type VII collagen alpha 1 chain. DEB can be inherited in an autosomal dominant (DDEB) or autosomal recessive (RDEB) pattern. Type VII collagen is synthesized and secreted as a procollagen by keratinocytes and fibroblasts, consisting of a globular N-terminus, a triple-helical collagenous domain (THC) and a C-terminus. Procollagen molecules first assemble into antiparallel dimers via C-terminal disulfide bonds; subsequent cleavage of the C-terminal propeptide by procollagen C-proteinase promotes the assembly of these dimers into anchoring fibrils, which form the lower component of the dermal-epidermal junction adhesion complex (5). As of March 21, 2026, the Human Gene Mutation Database^1^ has cataloged more than 800 *COL7A1* variants associated with DEB, including but not limited to missense, nonsense, splice-site, and deletion/insertion variants, with missense and nonsense variants being the most prevalent for this gene, followed by splice-site variants and small deletions. Previous studies demonstrated that variants in RDEB are distributed throughout the entire type VII collagen molecule, whereas those in DDEB are predominantly localized to the THC of *COL7A1* and are mostly glycine substitution variants (6). Therefore, most patients with RDEB present with more severe cutaneous manifestations and more frequent involvement of the digits and dentition than those with DDEB.

To the best of our knowledge, this is the first reported case of DEB-Pr harboring concurrent pathogenic variants in *COL7A1*, *CARD14*, and *G6PD*. A systematic literature search was performed in PubMed, Web of Science Core Collection, Embase, and Scopus (up to March 21, 2026) using the query: ((epidermolysis bullosa pruriginosa OR pruriginosa OR epidermolysis bullosa OR dystrophic epidermolysis bullosa) AND (COL7A1 AND CARD14 AND G6PD)). No prior reports of this unique variant combination in DEB-Pr were identified. The *COL7A1* p.Gly2254Arg variant, previously curated in the ClinVar database^2^ (7), maps to the triple-helical domain and results in the substitution of glycine (Gly) with arginine (Arg) at amino acid position 2,254 of the encoded protein. Studies have demonstrated that glycine substitutions within the collagen triple-helical domain destabilize the helix structure and cause functional impairment of anchoring fibrils in the sublamina densa of the cutaneous basement membrane zone (8). The patient developed clinical manifestations during adolescence with relatively localized cutaneous lesions and no involvement of the palms, soles, or nails. Combined with the pedigree analysis and genetic testing results, these features are consistent with autosomal dominant DEB (DDEB). Pathogenic variants in *CARD14* have been primarily associated with psoriasis vulgaris, psoriatic arthritis, generalized and palmoplantar pustular psoriasis, pityriasis rubra pilaris, and atopic dermatitis (9). However, the *CARD14* c.2172C>A (p.Tyr724*) variant identified in our patient is classified as a VUS, as it has been reported in healthy control cohorts but not in any patient cohorts to date (10). Therefore, this variant is not considered to contribute to the patient’s clinical phenotype or therapeutic response. The pathogenic *G6PD* c.1388G>A (p.Arg463His) variant explains the patient’s history of favism. No *G6PD* deficiency-related adverse events (e.g., hemolysis, aggravated anemia, or abnormal liver function) were observed during the 22-month upadacitinib treatment period. Although this finding is encouraging, given the single-case nature of this report, the long-term safety of upadacitinib in patients with *G6PD* deficiency requires further evaluation in large-scale cohort studies.

The pathogenic mechanism underlying pruritus in DEB-Pr remains incompletely elucidated, but recent studies have provided important mechanistic insights. Wu et al. demonstrated an increased proportion of Th2 cell subsets in the peripheral blood of patients with DEB-Pr, suggesting that type 2 inflammatory responses may contribute to the pathogenesis of this condition (11). This finding is further supported by tissue-level studies: recent investigations have shown increased expression of Th2-associated genes (e.g., *IL4R*, *CCL22*, *and SOCS3*) and proteins (e.g., IL-4, IL-13R) in cutaneous lesions of patients with DDEB. Bulk RNA sequencing and quantitative real-time PCR analysis have revealed that dupilumab-treated EB skin exhibits transcriptomic profiles similar to those of healthy skin, characterized by reduced enrichment of Th1/Th2 and Th17 signaling pathways—providing a theoretical basis for the use of dupilumab in the treatment of DDEB (12). To date, some case reports have demonstrated the efficacy of dupilumab in the treatment of EB (11, 13). In addition, elevated levels of multiple proinflammatory cytokines (e.g., IL-1β, IL-6, TNF-*α*, IFN-*γ*) have been detected in patients with DEB, indicating that immune dysregulation in this condition is not limited to the Th2 signaling pathway (12, 14). The JAK–STAT signaling pathway serves as a common downstream hub for signal transduction of multiple Th1, Th2, and Th17-associated cytokines (including IL-4 and IL-13). Thus, JAK inhibitors (JAKi) can theoretically inhibit the aberrant inflammatory network in DEB through a broad-spectrum mechanism. A retrospective case report has documented the efficacy of JAKi in the treatment of refractory pruritus in DEB (15). Real-world experience analyses have suggested that JAKi may offer advantages over dupilumab (which selectively targets IL-4/IL-13) in the management of pruritus in DEB-Pr, due to their ability to simultaneously inhibit multiple inflammatory pathways (e.g., Th1/Th17) (13).

This case is the first to describe a patient with DEB-Pr and a complex genetic background (concurrent *COL7A1*, *CARD14*, and *G6PD* variants) who achieved significant clinical improvement with oral upadacitinib following failure of topical corticosteroids and oral antihistamines. Rapid amelioration of cutaneous lesions and pruritus was observed within the first 3 months of treatment, and lesions continued to improve with no significant pruritus recurrence during gradual upadacitinib dose tapering. Ultimately, maintenance therapy with 15 mg once weekly was initiated at month 16. Although the patient experienced transient pruritus relapse and aggravated skin lesions after self-discontinuing treatment for 2 weeks at month 19, symptoms were rapidly controlled following treatment resumption, with no sustained disease recurrence thereafter up to the 22-month follow-up. This finding suggests that inhibition of the JAK–STAT signaling pathway may represent a potentially effective therapeutic strategy for refractory DEB-Pr, even in the presence of additional genetic modifying factors. Given the chronic, progressive nature of DEB and the unmet clinical need for effective treatments, the long-term safety and efficacy of JAKi require further investigation. Additional rigorous clinical studies (including prospective cohort studies and randomized controlled trials) are needed to validate these findings and establish comprehensive safety and dosing guidelines for the long-term use of JAKi in patients with DEB.

## Limitations

The findings of this study must be interpreted in the context of the current evidence base and the specific study limitations. Firstly, the existing clinical evidence for JAKi in the treatment of EB—including this case report—is primarily based on case reports and uncontrolled observational studies; randomized, double-blind, placebo-controlled clinical trial data are currently lacking. Secondly, the 22-month follow-up of this case provides valuable insights into the mid-term efficacy and safety of upadacitinib in DEB-Pr, but extended follow-up is essential for a comprehensive assessment of the long-term prognosis of this therapeutic approach in patients with this chronic mucocutaneous disorder. Although the significant clinical improvement and favorable tolerability observed in this case provide limited supportive evidence for the use of JAKi in patients with refractory DEB-Pr, future studies with more rigorous study designs and longer follow-up periods are required to confirm these preliminary findings.

## Conclusion

In summary, this case report describes a patient with refractory DEB-Pr harboring a unique combination of *COL7A1*, *CARD14*, and *G6PD* variants who achieved marked clinical improvement with oral upadacitinib treatment. Significant pruritus relief and cutaneous lesion healing were observed over a 22-month observation period, with no treatment-related adverse events. At present, there is no curative or definitive first-line treatment for DEB-Pr, but this case suggests that upadacitinib may represent a potentially effective and well-tolerated therapeutic option for patients with this refractory disease. However, these findings require validation in large-scale, controlled clinical trials with extended follow-up periods to firmly establish the efficacy, safety profile, and optimal dosing regimen of upadacitinib and other JAKi in the treatment of DEB-Pr.

## Data Availability

The original contributions presented in the study are publicly available. This data can be found at the National Genomics Data Centre under accession number HRA015858.

## Footnotes

1
https://www.hgmd.cf.ac.uk/ac/

2
https://www.ncbi.nlm.nih.gov/clinvar/
